# Plant occurrences on the Rybachy and the Sredny Peninsulas, Murmansk Region, Russia: a dataset

**DOI:** 10.3897/BDJ.10.e77094

**Published:** 2022-01-06

**Authors:** Ksenia Popova, Anna Razumovskaya

**Affiliations:** 1 Lomonosov Moscow State University, Moscow, Russia Lomonosov Moscow State University Moscow Russia; 2 Institute of Industrial Ecology Problems of the North (INEP KSC RAS), Apatity, Russia Institute of Industrial Ecology Problems of the North (INEP KSC RAS) Apatity Russia

**Keywords:** vascular plants, databasing, Barents Sea, Murmansk Region, Subarctic, plant distribution

## Abstract

**Background:**

The Rybachy and the Sredny Peninsulas are the northernmost part of Murmansk Region in the European part of Russia. While the most part of the Region is covered by boreal forest, the Peninsulas are covered by tundra. The vegetation and flora of Murmansk Region are well studied at present. The Peninsulas were first studied in 1829 by a Finnish botanist Jacob Fellman. The most comprehensive research was conducted in the late 19^th^ - early 20^th^ century. Nevertheless, the species composition of the Peninsulas' flora has changed significantly over the past 100 years due to land use and climate change. The aim of this dataset is to make the data on species occurrences for this territory digitally available via GBIF. To date, more or less complete digital floristic data were provided only by the project for digitising the book "Flora of Murmansk Region" (1953–1966).

The present dataset is a part of the project studying the vegetation of the territory. We recorded the information about species frequency and distribution using the relevé method.

**New information:**

We present a dataset based on 991 relevés from all vegetation types, which includes 16,289 records of georeferenced plant occurrences that belong to 568 species. There are 23 species of lichens (Ascomycota), 142 species of mosses (Bryophyta), three species of liverworts (Marchantiophyta) and 400 species of vascular plants (Tracheophyta) in the present dataset. The taxonomic diversity and unevenness result from the vegetation sampling. The data were collected in 2008, 2009, 2011, 2014 and 2015. The dataset cannot be considered as a complete vegetation database or a flora checklist, but it contains the occurrences and frequencies of the species from all the vegetation types.

## Introduction

The contemporary studies of biodiversity and biogeography require digital, available, relevant and detailed data (König et al. 2019). GBIF — the Global Biodiversity Information Facility — was established to accumulate these data 20 years ago. Although this network has been in existence long enough, the data from Russia are still poor. Even Murmansk Region, a well-studied area compared with other regions of Russia, has only about 200,000 occurrences of species in GBIF. For example, Norway and Finland, whose areas are a little more than twice the size of Murmansk Region, have 130-200 times more occurrences than Murmansk Region (according to GBIF data).

For our study, we chose the north-westernmost part of the Region - the Rybachy and the Sredny Peninsulas. This territory has a relatively high biodiversity and is valuable in terms of nature conservation.

**Figure 1.**
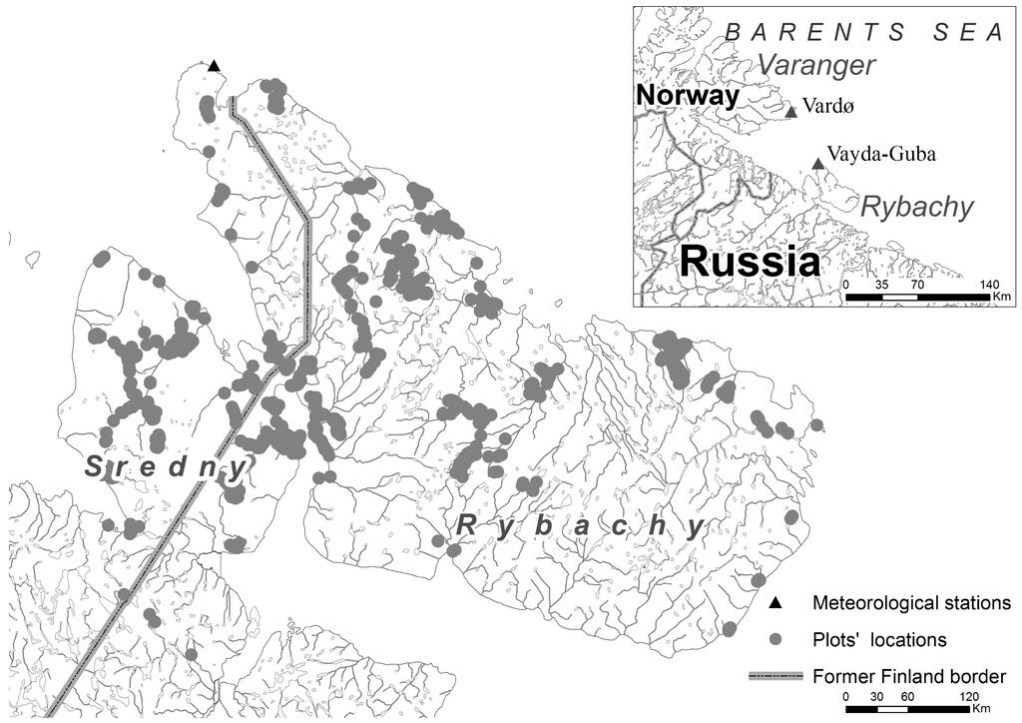
Map of the Rybachy and the Sredny Peninsulas with locations of vegetation plots and the meteorological stations closest to the study site (inlay).

The Peninsulas were first studied in 1829 by a Finnish botanist Jacob Fellman (Uotila 2013). The most comprehensive study of the Peninsulas' vegetation was published by Aarno Kalela (1939). He studied herbaceous plant communities in the western part of the Peninsulas, which, at that time (1927–1930), belonged to Finland (Fig. 1). The Rybachy and the Sredny Peninsulas were then used for fishing and (semi-)domestic livestock husbandry. Afterwards, the land-use activity has stopped and the climate has changed significantly. As a result, the species composition and species richness have also changed (Kapfer and Popova 2020).

The flora and vegetation of the western part of Murmansk Region were most thoroughly studied in the late 19^th^ - early 20^th^ centuries (Kozhin et al. 2020b). These observations were collected in "Flora of Murmansk Region" (published in 1953–1966) and then digitised (Kozhin et al. 2020a). This database contains about 1,500 occurrences of vascular plants from the Peninsulas' area.

The largest occurrence dataset of cryptogam organisms in Russia is CRIS (Melekhin et al. 2019). The majority of records in CRIS are from Murmansk Region, but this dataset includes only 357 observations for the Rybachy and the Sredny Peninsulas (Cris and Melechin 2019).

Thus, more digital data on the distribution of plants in the study area are still required for a better coverage in GBIF.

## General description

### Purpose

Our dataset contributes to studying plant species distribution (mostly vascular) on the Rybachy and the Sredny Peninsulas (Popova and Razumovskaya 2021) and to updating the information about species of conservation concern and rare species.

### Additional information

This work is a spin-off project of our long-term studies of the vegetation of the Peninsulas. A total of 991 relevés were compiled using the Braun-Blanquet approach (Westhoff and Van Der Maarel 1978) and then classified. The rank of the classes follows the last survey on the European vegetation (Mucina et al. 2016).

The preliminary results suggest the existence of 20 vegetation classes (Popova 2019):

1. ***Loiseleurioprocumbentis*-*Vaccinietea*** Eggler ex Schubert 1960,
2. ***Caricirupestris*-*Kobresieteabellardii*** Ohba 1974,
3. ***Vaccinio*-*Piceetea*** Br.-Bl. in Br.-Bl. et al. 1939,
4. ***Saliceteaherbaceae*** Br.-Bl. 1948,
5. ***Junceteatrifidi*** Hadač in Klika et Hadač 1944,
6. ***Mulgedio*-*Aconitetea*** Hadač et Klika in Klika et Hadač 1944,
7. ***Molinio*-*Arrhenatheretea*** Tx. 1937,
8. ***Scheuchzeriopalustris*-*Cariceteafuscae*** Tx. 1937,
9. ***Oxycocco*-*Sphagnetea*** Br.-Bl. et Tx. ex Westhoff et al. 1946,
10. ***Betulocarpaticae*-*Alneteaviridis*** Rejmánek ex Bœuf, Theurillat, Willner, Mucina et Simler in Bœuf et al. 2014,
11. ***Ammophiletea*** Br.-Bl. et Tx. ex Westhoff et al. 1946,
12. ***Cakileteamaritimae*** Tx. et Preising in Tx. ex Br.-Bl. et Tx. 1952,
13. ***Junceteamaritimi*** Br.-Bl. in Br.-Bl. et al. 1952,
14. ***Littorelleteauniflorae*** Br.-Bl. et Tx. ex Westhoff et al. 1946,
15. ***Montio*-*Cardaminetea*** Br.-Bl. et Tx. ex Klika et Hadač 1944,
16. ***Phragmito*-*Magnocaricetea*** Klika in Klika et Novák 1941,
17. ***Asplenieteatrichomanis*** (Br.-Bl. in Meier et Br.-Bl. 1934) Oberd. 1977,
18. ***Racomitrieteaheterostichi*** Neumayr 1971,
19. ***Polygono*-*Poeteaannuae*** Rivas-Mart. 1975,
20. ***Epilobieteaangustifolii*** Tx. et Preising ex von Rochow 1951.

Thus, our data cover the whole diversity of plant communities on the Rybachy and the Sredny Peninsulas. Most of the species in the dataset are diagnostic for different syntaxa (Mucina et al. 2016). Thus, the data can be used for modelling the distribution of both the plant species and their communities.

## Sampling methods

### Study extent

The Rybachy and the Sredny Peninsulas are situated at the 69^th^ latitude and washed by the Barents Sea (Fig. 1). The study area is within the zone of Subarctic Tundra (Aleksandrova 1977).

Geological structure. The Peninsulas' bedrocks consist of upper Proterozoic solid rocks, such as sandstones, shales and conglomerates. It makes them different from the mainland coast of Murmansk Region, which generally consists of granites and granitoids. Pre-Quaternary deposits form the most part of Rybachy Peninsula's coast. The Peninsulas' inland is built up with glacial deposits of the Last Glacial Period, which are mainly sandy loam and loamy with rubble or boulders with lenses and interlayers of sand and clay. The Rybachy and the Sredny Peninsulas are mostly similar to the Varanger Peninsula (Finnmark, Norway) due to the geological structure of the bedrocks (Siedlecka and Siedlecki 1967).

Landscape. The inland high plains rise up to 200-300 m. The coast is abrasional and has the pediment plain up to 50 m in altitude (Miloserdov 1971) with a series of accumulative marine terraces well developed in bays.

Climate. The climate is oceanic, influenced by the southern extension of the Nord Cape stream making it milder than the climate of the Kola Peninsula. According to the data received from the Vaida-Guba (Fig. 1) weather station for the last 10 years, the mean annual temperature is +2.4°С (calculated on the data from www.rp5.ru). During the last 100 years, the mean annual temperature has increased by 1.2°C (Kapfer and Popova 2020). The annual precipitation is about 700 mm and has changed twice for the last ca. 100 years. The vegetation period (the time when the daily mean temperature is more than +5°С) lasts for more than 100 days (Miloserdov 1971). The depth of the snow cover does not exceed 40 cm. During the last 10 years, it was up to 18 cm (calculated on the data from www.rp5.ru).

Soil cover. The Peninsulas are mainly covered by podzols rich in aluminium (Al) and iron (Fe). The layers cannot be well-defined due to the bedrock structure. Permafrost is not spread in the area, but it can be found in some spots (Koroleva and Pereversev 2007).

Vegetation. The Peninsulas are mostly covered by dwarf shrub tundra with the dominance of Empetrumnigrumssp.hermaphroditum, *Vacciniumvitis-idaea*, *Vacciniumuliginosum* and *Vacciniummyrtillus*. The heterogeneity of the terrain and the abundance of small rivers and lakes create a lot of microsites for different types of vegetation. The most common plant communities are dwarf shrub tundra, birch krummholz, willow-shrub communities, bogs, fens, meadows, grasslands on sand dunes, salt-marshes, chasmophytic vegetation of rocks, vegetation of scree habitats and ruderal communities of trampled habitats.

The Peninsulas are not the only location covered by tundra communities in Murmansk Region, but they differ from other parts of the Region in some features of geological structure, climate and land use. Most of all, this territory can be regarded as a model of the vegetation modification in the Arctic following the climate change.

The protected area "The Rybachy and the Sredny Peninsulas" was created on the largest part of the Peninsulas in 2014. This area has a status of regional Natural Park. Our vegetation and flora studies started as part of a nature conservation project and then continued to adjust the protection regime, to catalogue the vegetation cover and to identify the vegetation development patterns.

### Sampling description

The data were collected using the vegetation plot method as described below; the observations are located irregularly reflecting the habitat distribution. In the summers of 2008, 2009, 2011, 2014 and 2015, the authors of this paper sampled 991 vegetation plots (relevés). For each relevé, we recorded the cover of the layers and the cover of each species, the relevé area size, slope aspect and slope ratio and height of each vegetation layer. The plot sizes varied due to the size of the plant communities. For tundra and bog communities, the size of the plots was 400 m^2^ (20 × 20 m). The other communities were often less than 400 m^2^ in area; thus, the relevés were made within the boundaries of the plant community. In this case, the plot size varied from 1 m^2^ to 400 m^2^. In the current dataset, we do not present this information. We also recorded the location of the plots (geographical coordinates in WGS84). The sampling process was conducted with direct observations and active search for plant species, i.e. vascular plants, mosses, liverworts and lichens.

The vegetation database was created using the Turboveg software (Hennekens and Schaminée 2001). The database was converted into the Darwin Core format (Wieczorek et al. 2012) for integration with GBIF. All supporting information (i.e. ecological information and covers) was deleted. The duplicates (the same species with the same coordinates) were detected and also deleted. The dataset was hosted by the Moscow State University.

### Quality control

All the taxa in the dataset have the rank "species". We did not include the specimens, which were not identified at the species rank. The plant names are provided according to Cherepanov (1995) (vascular plants), Ignatov et al. (2006)(mosses), Urbanavichus (2010) (lichens) and Potemkin and Kotkova (2019) (liverworts).

Species difficult for field identification were collected for further work and the specimens were identified by the authors. The moss herbarium specimens, collected in 2008, 2009 and 2011, were included in the dataset. Identifications of mosses were made and/or confirmed by E. A. Ignatova and V. E. Fedosov (Lomonosov Moscow State University). Herbarium specimens of problematic vascular plant taxa were checked by experts from several institutions: Komarov Botanical Institute - N. N. Tzvelev (Poaceae, *Sparganium*, *Potomogeton*), V. I. Dorofeev (Brassicaceae), V. V. Petrovskyi (*Draba*), T. V. Egorova (*Carex*), A. E. Grabovskaya-Borodina (*Rumex*), G. Yu. Konechnaya (various taxa), I.B. Kucherov (*Alchemilla*); Lomonosov Moscow State University - M.N. Kozhin (*Betula*, *Atriplex*); Polar-Alpine Botanical Garden-Institute - V. A. Kostina (specimens collected in 2008). The specimens after identification and revision were deposited to the Herbaria of the Moscow State University (MW), Institute of North Industrial Ecology Problems (INEP), Karelian Research Centre (PTZ) and Komarov Botanical Institute (LE).

This dataset cannot be used as a vegetation database or a floristic checklist. We did not provide the information about species coverage and height and ecological conditions (terrain and soils).

## Geographic coverage

### Description

The study area is located in the northern part of Pechengsky District of Murmansk Region, representing the northernmost part of continental European Russia (69°33′–69°56′ N, 31°44′–32°07′ E; Fig. 1). The study site is divided into two parts: the smaller southern part, the Sredny Peninsula and the larger northern part, the Rybachy Peninsula. The Sredny Peninsula is connected with the Rybachy by a 2-km-long isthmus in the north and separated from the mainland by a 4-km-wide fell – Mustatunturi Mountain.

The majority of occurrences are located in the Rybachy Peninsula (11,346, 70%). The Sredny Peninsula is covered by 4,536 occurrences (28%). The isthmus between the Peninsulas is represented by 133 occurrences (less than 1%). We also included several observations (274, 1%), located at the Mustatunturi Mt. nearby the Sredny Peninsula.

The locations of the occurrences are not regularly situated. By choosing the location of a vegetation plot, we tried to reveal the alpha, beta and gamma diversity to the fullest extent possible. Thus, the inland part of the Rybachy Peninsula, which is homogeneous in vegetation and not rich in species diversity, is covered by a fewer number of observations than the river valleys and the sea coasts.

The southeast part of the Rybachy Peninsula remains less studied than the rest of the territory, because it is hard to reach from both the land (due to the absence of roads) and the sea (due to the cliffs).

### Coordinates

E31,8758° and E31,9367° Latitude; N69,7244° and N69,7182° Longitude.

## Taxonomic coverage

### Description

Our dataset includes 16,289 records of georeferenced plant occurrences that belong to 568 species. There are 23 species of lichens (Ascomycota), 142 species of mosses (Bryophyta), three species of liverworts (Marchantiophyta) and 400 species of vascular plants (Tracheophyta) in the presented dataset.

Since our data (Popova and Razumovskaya 2021) are based on vegetation plots, they contain vascular plants, mosses, liverworts and lichens. The data on lichens, mosses and liverworts were included in the dataset because these groups are represented by few occurrences in GBIF. For example, the CRIS dataset (Cris and Melechin 2019) contains only 357 occurrences for the Peninsulas' territory, amongst them 339 occurrences of fungi (including lichens), 13 for liverworts and only one for mosses. We do not provide a complete list of mosses, liverworts and lichens. Our data include only the most frequent species. The lichens and liverworts species in our dataset represent about 2% and 1.5% of the total diversity in the Region, respectively (Melechin 2009, Konstantinova et al. 2009). Our data cover only about 30% of the total diversity of mosses or vascular plants in Murmansk Region (Belkina et al. 2009, Kostina and Fillimonova 2009).

Based on our estimates of the diversity of mosses and vascular plants on the Peninsulas (yet unpublished), the dataset covers approximately 80% of vascular plant species and about 50% of moss species on the Rybachy and the Sredny Peninsulas.

The majority of occurrences belong to vascular plants (Table 1). The most frequent species (with more than 300 observations) are Empetrumnigrumsubsp.hermaphroditum, *Betulanana*, *Solidagovirgaurea* and *Avenellaflexuosa*. The most species-rich families are Poaceae (54), Cyperaceae (36), Caryophyllaceae (28), Rosaceae (24) and Asteraceae (22).

Although the data on vascular plants are relatively complete, they do not include several most difficult taxa. The majority of *Alchemilla* species still remain unidentified; the dataset includes only determined species of this genus. In apomictic genera (*Taraxacum*, *Hieracium)*, we included only the species easy to identify: *Taraxacumofficinale* (s.l.) and *Hieraciumumbellatum*. Moreover, the dataset does not include the occurrences for several *Ranunculus*, *Betula*, *Salix* and *Euphrasia* species, which were observed at the plots, but remain unidentified.

**Figure 2.**
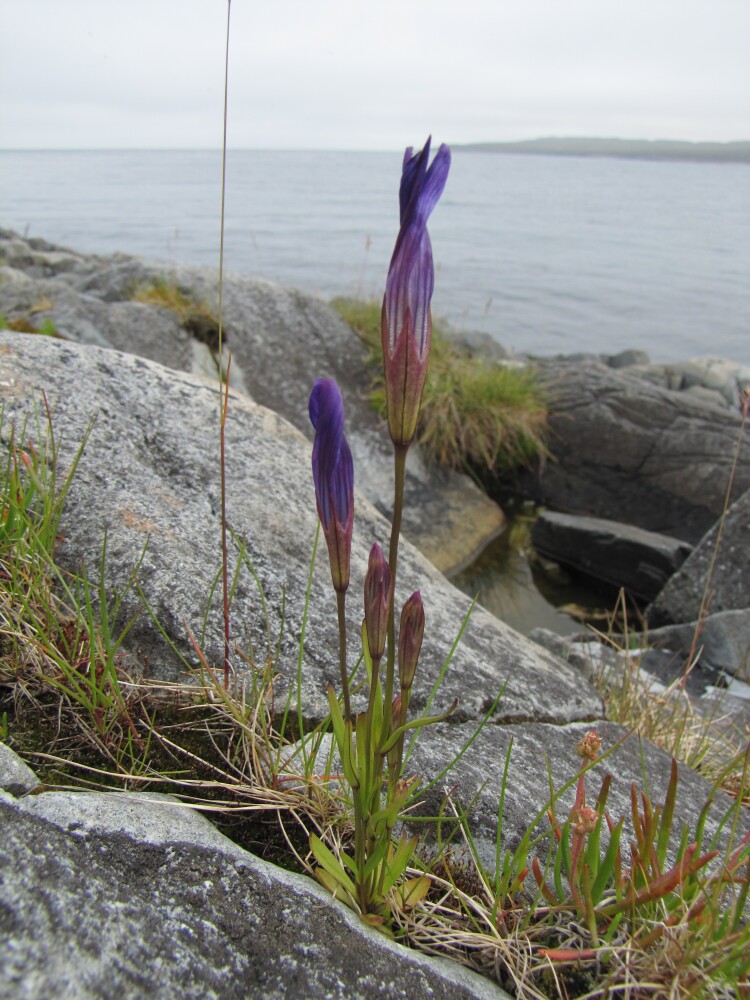
The rare species *Gentianopsisdetonsa* (Rybachy Peninsula, photo by K. Popova, July 2011).

**Figure 3.**
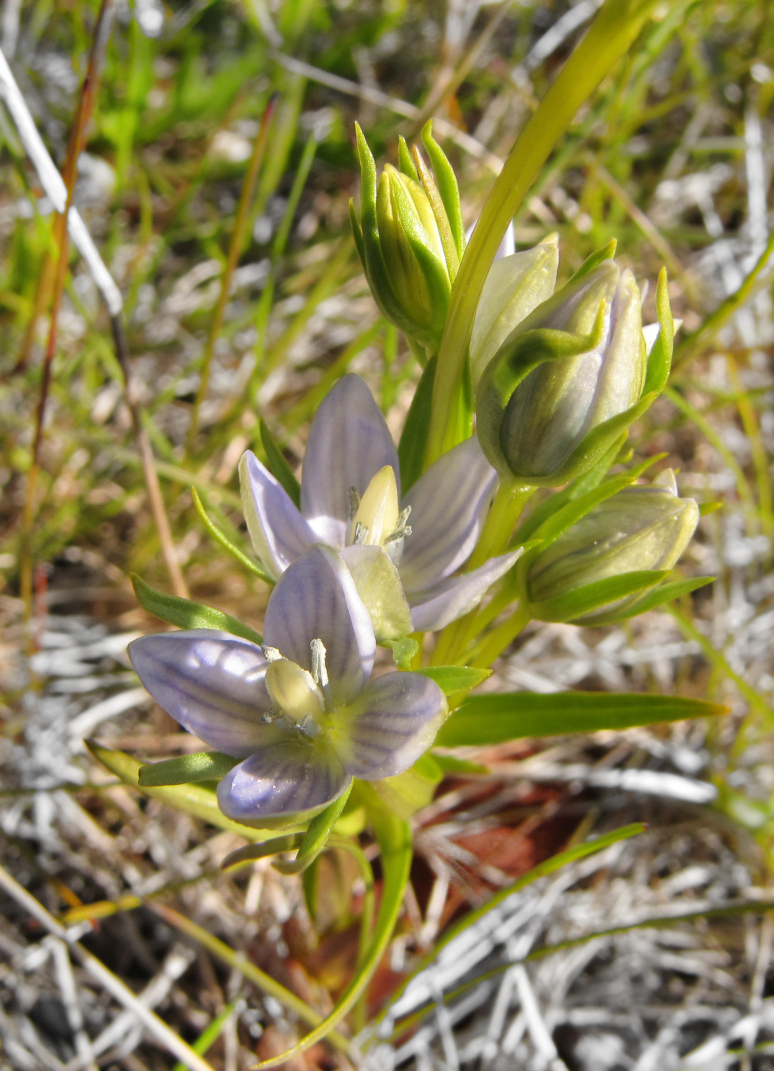
The rare species *Lomatogoniumrotatum* (Rybachy Peninsula, photo by K. Popova, July 2011).

**Figure 4.**
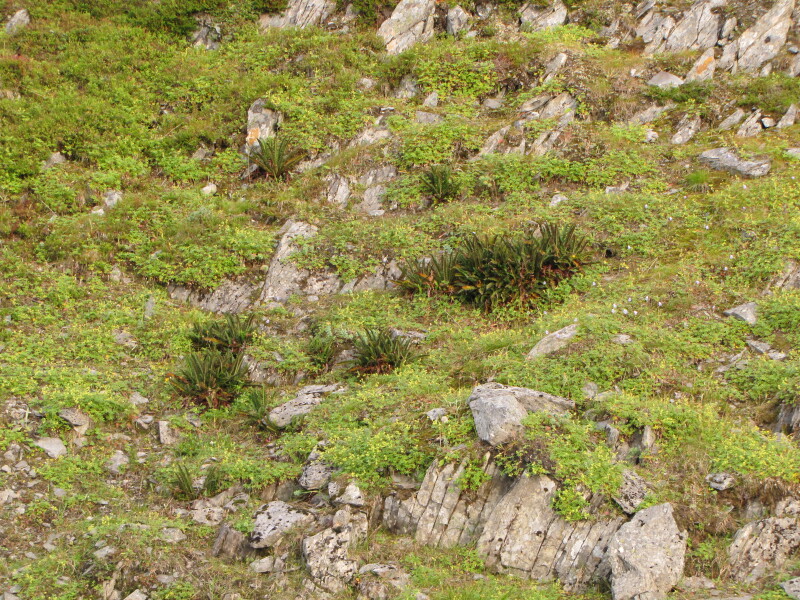
Plant community dominated by two Red List species, *Alchemillaalpina* and *Polystichumlonchitis* (Rybachy Peninsula, photo by K. Popova, August 2014).

**Figure 5.**
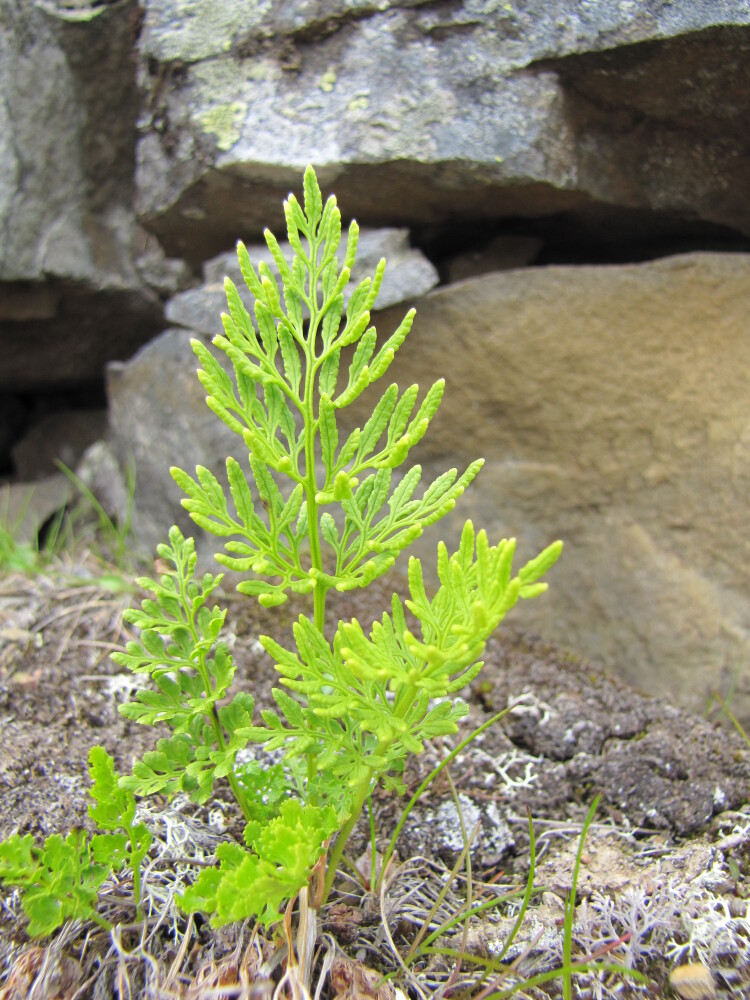
The rare species *Cryptogrammacrispa* (Sredny Peninsula, photo by K. Popova, August 2015).

The dataset includes the occurrences of legally protected species (Table 2). Some of them are really rare in Murmansk Region and known only by few (or even one) localities. These are *Eritrichiumvillosum* (1 locality in the Region: Kozhin et al. 2020b, Borovichev et al. 2019), *Gastrolychnisapetala* (2 localities in the Region), *Gentianopsisdetonsa* (4 localities in the Region, Fig. 2), *Lomatogoniumrotatum* (2 localities in the Region, Fig. 3) and *Drabafladnizensis* (3 localities in the Region). The number of localities is provided according to the Red Data Book of Murmansk Region (Konstantinova et al. 2014). Not least important are the occurences of the species, which are rare in Murmansk Region and in Russia, but are common in the Rybachy Peninsula or the Sredny Peninsula. One of such species is *Alchemillaalpina* (*Fig. 4*). It is a typical and usually dominant species of screes, roadsides, snowbeds and meadows on the Rybachy Peninsula. The second species is *Cryptogrammacrispa* (Fig. 5), which has a large population on rocks in the Sredny Peninsula.

The dataset contains the up-to-date information about the distribution of some rare species, which were observed in Murmansk Region only a few times and more than 50 years ago, for example, *Gastrolychnisapetala*, *Eritrichiumvillosum*, *Gentianopsisdetonsa* (Fig. 2) and *Lomatogoniumrotatum* (Fig. 3).

## Temporal coverage

**Data range:** 2008-8-08 – 2015-7-30.

### Notes

In the summers of 2008, 2009, 2011, 2014 and 2015, we studied the vegetation cover of the Peninsulas. The longest period of fieldwork was in 2014, so most of the occurrences (9,263) were recorded during that year. The dataset additionally contains 1,557 occurrences made in 2008, 961 - in 2009, 311 - in 2011 and 4,197 - in 2015. Such differences in the number of observations are a consequence of different durations of the field seasons.

## Usage licence

### Usage licence

Creative Commons Public Domain Waiver (CC-Zero)

## Data resources

### Data package title

Vegetation of the Rybachy Peninsula and the Sredny Peninsula, Murmansk Oblast, Russia

### Resource link

https://www.gbif.org/dataset/c1d45286-a9a3-4399-acf3-1eccb8c68673

### Alternative identifiers

https://doi.org/10.15468/87k7q3

### Number of data sets

1

### Data set 1.

#### Data set name

Vegetation of the Rybachy Peninsula and the Sredny Peninsula, Murmansk Oblast, Russia

#### Number of columns

46

#### Description

The dataset presents the occurrences of the 568 species of plants on the Rybachy and the Sredny Peninsulas (NW Russia), based on original vegetation plots.

**Data set 1.** 

Column label	Column description
occurrenceID	An identifier for the Occurrence (ID of a record within the dataset). For example, "13760".
dcterms:type	The nature or genre of the resource. A constant ("Dataset").
dcterms:modified	The most recent date-time on which the resource was changed.
dcterms:language	A language of the resource. A constant ("en", i.e. English).
dcterms:license	A legal document giving official permission to do something with the resource. A constant ("http://creativecommons.org/licenses/by/4.0/legalcode").
dcterms:rightsHolder	A person or organisation owning or managing rights over the resource. A constant ("Moscow State University").
dcterms:accessRights	Information about who can access the resource or an indication of its security status. A constant ("Use under CC BY 4.0").
institutionID	An identifier for the institution having custody of the object(s) or information referred to in the record. A constant ("http://grbio.org/institution/moscow-stateuniversity" for the Moscow State University).
collectionID	An identifier for the collection or dataset from which the record was derived. A constant ("urn:lsid:biocol.org:col:15550" for the Moscow University Herbarium).
datasetID	An identifier for the set of data. May be a global unique identifier or an identifier specific to a collection or institution. A constant ("urn:lsid:biocol.org:col:15550:09").
institutionCode	The name (or acronym) in use by the institution having custody of the object(s) or information referred to in the record. A constant ("Moscow State University").
datasetName	The name identifying the dataset from which the record was derived. A constant (Vegetation of the Rybachy Peninsula and the Sredny Peninsula, Murmansk Oblast, Russia).
ownerInstitutionCode	The name (or acronym) in use by the institution having ownership of the object(s) or information referred to in the record. A constant ("Moscow State University").
basisOfRecord	The specific nature of the data record – a subtype of the dcterms:type. A constant ("HumanObservation").
informationWithheld	Additional information that exists, but that has not been shared in the given record. A constant ("Associated ecological data").
catalogNumber	An identifier (preferably unique) for the record within the dataset or collection. A variable. The same with “occurrenceID”.
recordedBy	A list of names of people responsible for recording the original Occurrence. A variable. For example, “Anna Razumovskaya | Ksenia Popova”.
occurrenceStatus	A statement about the presence or absence of a taxon at a location. A constant ("present").
eventID	An identifier for the set of information associated with an Event (something that occurs at a place and time). Vegetation plot number, relevé number. A variable. For example, “869”.
eventDate	The date or interval during which an event occurred. For occurrences, this is the date when the event was recorded. A variable. For example, “2015-07-10”.
day	The integer day of the month on which the Event occurred. A variable.
month	The integer month in which the Event occurred. A variable.
year	The four-digit year in which the Event occurred, according to the Common Era Calendar. A variable.
higherGeography	A list (concatenated and separated) of geographic names less specific than the information captured in the locality term. A constant ("Europe | Russian Federation | Murmansk Oblast | Pechengsky District").
continent	The name of the continent in which the location occurs. A constant ("Europe").
country	The name of the country or major administrative unit in which the location occurs. A constant ("Russian Federation").
countryCode	The standard code for the country in which the location occurs. A constant ("RU").
stateProvince	The name of the next smaller administrative region than country (state, province, canton, department, region etc.) in which the location occurs. A constant ("Murmansk Oblast").
county	The full, unabbreviated name of the next smaller administrative region than stateProvince (county, shire, department etc.) in which the Location occurs. A constant ("Pechengsky District").
decimalLatitude	The geographic latitude (in decimal degrees, using the spatial reference system given in geodeticDatum) of the geographic centre of a location. A variable.
decimalLongitude	The geographic longitude (in decimal degrees, using the spatial reference system given in geodeticDatum) of the geographic centre of a location. A variable.
geodeticDatum	The ellipsoid, geodetic datum or spatial reference system (SRS) upon which the geographic coordinates given in decimalLatitude and decimalLongitude are based. A constant ("WGS84").
coordinateUncertaintyInMeters	The horizontal distance (in metres) from the given decimalLatitude and decimalLongitude describing the smallest circle containing the whole of the location. A constant (“10”)
georeferencedBy	A list (concatenated and separated) of names of people, groups or organisations who determined the georeference (spatial representation) of the location. The same with “recordedBy”.
scientificName	The full scientific name, with authorship and date information, if known. A variable (for example, "Epilobium hornemannii Rchb.").
genus	The full scientific name of the genus in which the taxon is classified. A variable (for example, "Epilobium").
specificEpithet	The name of the first or species epithet of the scientificName. A variable (for example, "hornemannii").
scientificNameAuthorship	The authorship information for the scientificName formatted according to the conventions of the applicable nomenclaturalCode. A variable (for example, "Rchb.").
nomenclaturalCode	The nomenclatural code (or codes in the case of an ambiregnal name) under which the scientificName is constructed. A constant ("International Code of Nomenclature for algae, fungi and plants").
taxonomicStatus	The status of the use of the scientificName as a label for a taxon. A constant ("accepted").
taxonRank	The taxonomic rank of the most specific name in the scientificName. A constant ("species").
kingdom	The full scientific name of the kingdom in which the taxon is classified.
phylum	The full scientific name of the phylum or division in which the taxon is classified.
class	The full scientific name of the class in which the taxon is classified.
order	The full scientific name of the order in which the taxon is classified.
family	The full scientific name of the family in which the taxon is classified.

## Figures and Tables

**Table 1.** Taxonomic distribution of occurrences.

Taxa	Species	Occurrences
Plantae		
**Tracheophyta**	400	14 395
Magnoliopsida	248	9 724
Liliopsida	122	3 825
Polypodiopsida	21	584
Lycopodiopsida	7	198
Pinopsida	2	64
**Bryophyta**	142	1 283
**Marchantiophyta**	3	79
Fungi		
**Ascomycota**	23	532

**Table 2.** The species under conservation concern in the provided dataset. Correspondence between conservation status in the Red Data Book of Murmansk Region (Konstantinova et al. 2014) and IUCN Red List Categories (IUCN 2012): 1a – Critically endangered (CR), 1b – Endangered (EN), 2 – Vulnerable (VU), 3 – Near threatened (NT)

Species	Number of occurrences in the dataset	Family	Conservation status in the Red Data Book of Murmansk Region
*Epilobiumalsinifolium* Vill.	1	Onagraceae	3
*Gastrolychnisapetala* (L.) Tolm. & Kozhanch.	1	Caryophyllaceae	3
*Eritrichiumvillosum* (Ledeb.) Bunge	1	Boraginaceae	1a
*Gentianopsisdetonsa* (Rottb.) Ma	1	Gentianaceae	1b
*Lomatogoniumrotatum* (L.) Fr. ex Fernald	1	Gentianaceae	1b
*Carexmaritima* Gunnerus	2	Cyperaceae	3
*Drabafladnizensis* Wulfen	2	Brassicaceae	3
*Armeriascabra* Pall. ex Roem. & Schult.	3	Plumbaginaceae	3
*Sphagnumsubnitens* Russow & Warnstorf	4	Sphagnaceae	3
*Polystichumlonchitis* (L.) Roth	6	Dryopteridaceae	3
*Cryptogrammacrispa* (L.) R.Br.	10	Pteridaceae	3
*Leucorchisalbida* (L.) E.Mey.	11	Orchidaceae	2
*Gentianellaaurea* (L.) H.Sm.	21	Gentianaceae	3
*Valerianasambucifolia* J.C.Mikan	21	Caprifoliaceae	3
*Alchemillaalpina* L.	86	Rosaceae	3
*Rhodiolarosea* L.	140	Crassulaceae	3
